# The inhibitory effect of Gremlin-2 on adipogenesis suppresses breast cancer cell growth and metastasis

**DOI:** 10.1186/s13058-023-01732-2

**Published:** 2023-10-25

**Authors:** Jiwoo Jung, Na Hui Kim, Minji Kwon, Jayeon Park, Dayeon Lim, Youjin Kim, World Gil, Ye Hwang Cheong, Sin-Aye Park

**Affiliations:** 1 Department of Medical Sciences, Graduate School, Soonchunhyang University, Asan-si, 31538 Republic of Korea; 2 Department of Biomedical Laboratory Science, College of Medical Sciences, Soonchunhyang University, Asan-si, 31538 Republic of Korea; 3 Drug Discovery Research Laboratories, Dong-A ST Co., Ltd., Yongin, 17073 Republic of Korea

**Keywords:** Gremlin-2, 3T3-L1 cells, Adipocytes, IL-6, Breast cancer cells, Cancer progression, Metastasis

## Abstract

**Background:**

Gremlin-1 (GREM1) and Gremlin-2 (GREM2) are bone morphogenetic protein antagonists that play important roles in organogenesis, tissue differentiation, and tissue homeostasis. Although GREM1 has been reported to be involved in promoting various cancers, little has been reported about effects of GREM2 on cancer. Recently, it has been reported that GREM2 can inhibit adipogenesis in adipose-derived stromal/stem cells. However, as an inhibitor of adipogenesis, the role of GREM2 in cancer progression is not well understood yet.

**Methods:**

Pre-adipocyte 3T3-L1 cells overexpressing mock or *Grem2* were established using a lentiviral transduction system and differentiated into adipocytes-mock and adipocytes-Grem2, respectively. To investigate the effect of adipocyte-Grem2 on breast cancer cells, we analyzed the proliferative and invasion abilities of spheroids using a 3D co-culture system of breast cancer cells and adipocytes or conditioned medium (CM) of adipocytes. An orthotopic breast cancer mouse model was used to examine the role of adipocytes-Grem2 in breast cancer progression.

**Results:**

*Grem2* overexpression suppressed adipogenesis of 3T3-L1 cells. Proliferative and invasion abilities of spheroids formed by co-culturing MTV/TM-011 breast cancer cells and adipocytes-Grem2 were significantly reduced compared to those of spheroids formed by co-culturing MTV/TM-011 cells and adipocytes-mock. Compared to adipocytes-mock, adipocytes-Grem2 showed decreased mRNA expression of several adipokines, notably IL-6. The concentration of IL-6 in the CM of these cells was also decreased. Proliferative and invasive abilities of breast cancer cells reduced by adipocytes-Grem2 were restored by IL-6 treatment. Expression levels of vimentin, slug, and twist1 in breast cancer cells were decreased by treatment with CM of adipocytes-Grem2 but increased by IL-6 treatment. In orthotopic breast cancer mouse model, mice injected with both MTV/TM-011 cells and adipocytes-Grem2 showed smaller primary tumors and lower lung metastasis than controls. However, IL-6 administration increased both the size of primary tumor and the number of metastatic lung lesions, which were reduced by adipocytes-Grem2.

**Conclusions:**

Our study suggests that *GREM2* overexpression in adipocytes can inhibit adipogenesis, reduce the expression and secretion of several adipokines, including IL-6, and ultimately inhibit breast cancer progression.

**Supplementary Information:**

The online version contains supplementary material available at 10.1186/s13058-023-01732-2.

## Background

Cysteine knot-secreting proteins Gremlin-1 (GREM1) and Gremlin-2 (GREM2) are bone morphogenetic protein (BMP) antagonists. These gremlins play important roles in embryogenesis, organ development, and tissue differentiation through antagonistic regulation of BMPs [1, 2]. GREM1 is implicated in various pathological conditions such as pulmonary or renal fibrosis [3–5] and diabetic kidney disease [6]. In addition, GREM1 is overexpressed in various cancers. It has been reported to be involved in cancer proliferation and metastasis [7, 8]. Unlike GREM1, GREM2 has few reports in these pathological conditions. GREM2 can inhibit the differentiation of bone marrow-derived mesenchymal stem cells into osteoblasts [9]. GREM2 can regulate cardiac differentiation of cardiac progenitor cells and enhance cardioprotective effect [10]. GREM2 plays a role in suppressing side effects of excessive inflammation after myocardial infarction by inhibiting BMP signaling [11]. More interestingly, GREM2 not only inhibits adipogenesis in 3T3-L1 pre-adipocytes [12], but also functions as an inhibitor of adipogenesis in adipose-derived stromal/stem cells [13] through activation of Wnt/β-catenin signaling. However, the role of GREM2 in cancer progression is not well understood yet.

Adipocytes are derived from pluripotent mesenchymal stem cells, converted into pre-adipocytes, and then differentiated into mature adipocytes. This process of differentiation of adipocytes is called adipogenesis. Various proteins such as peroxisome proliferator-activated receptor γ (PPARγ), CCAAT/enhancer binding protein α (C/EBPα), and fatty acid binding protein 4 (FABP4) are involved in adipogenesis [14–16]. Adipocytes are one of the major stromal cells of many tissues. They are considered to play an important role in promoting cancer growth in the tumor microenvironment [17]. In particular, since adipocytes are main stromal cells of breast tissues, interactions between adipocytes and breast cancer cells are highly correlated with breast cancer progression [18].

Cancer-associated adipocytes (CAAs) are involved in breast tumorigenesis [19]. CAAs secrete a variety of inflammatory and cancer-related adipokines such as tumor necrosis factor-α, interleukin-6 (IL-6), and plasminogen activator inhibitor-1 that can increase breast cancer cell proliferation, invasion, metastasis, and therapeutic resistance [20–23]. In particular, IL-6 and its associated signaling can promote breast cancer progression and metastasis [24–27]. IL-6 secreted from adipose stromal cells (ASCs) can promote the migration and invasion of estrogen receptor-negative breast cancer cells both in vitro and in vivo, while depletion of IL-6 in conditioned medium (CM) of ASCs can suppress the stimulatory effect of ASCs on breast cancer cell migration and invasion [28]. Adipocyte-derived IL-6 and leptin can promote breast cancer metastasis by promoting the expression of lysyl hydroxylase [24]. Serum IL-6 levels are positively correlated with serum VEGF levels in breast cancer patients, which can promote angiogenesis and metastasis [29]. IL-6 can increase resistance of breast cancer cells to drug treatment by inducing *Mdr1* gene expression [30], and downregulation of IL-6 is associated with better response to breast cancer treatment [31].

Inhibiting adipocyte differentiation or blocking adipokine secretion through modulation of adipocytes could be a new treatment paradigm for breast cancer. TAZ knockdown or deficiency in mouse adipocytes inhibits breast tumorigenesis through impaired expression and secretion of the adipocytes-derived hormone Resistin [32]. More recently, BZ26, a derivative of the PPARγ antagonist GW9662, attenuated breast cancer progression by inhibiting the progression of mature adipocytes into CAA-like cells [33]. Although many studies have reported that adipogenesis can promote breast cancer, studies on new adipogenesis inhibitors that can inhibit breast cancer growth and their mechanisms are not yet well known. In this study, we found that GREM2 suppressed adipocyte differentiation and that adipocytes overexpressing *Grem2* reduced the production of several adipokines, including IL-6, contributing to the inhibition of breast cancer cell growth and lung metastasis. These results suggest that overexpressing *GREM2* in adipocytes can be a new therapeutic approach to effectively inhibit breast cancer proliferation and metastasis.

## Methods

### Cell culture and reagents

3T3-L1 cells were purchased from the American Type Culture Collection (USA). 3T3-L1 cells were maintained in DMEM (Thermo Fisher Scientific, USA), bovine calf serum (BCS, HyClone, USA) and 1% penicillin/streptomycin (Corning Inc., USA). MDA-MB-231 and MTV/TM-011 cells were obtained from Korean Cell Line Bank (Republic of Korea). MDA-MB-231 and MTV/TM-011 cells were cultured in RPMI (Corning Inc.) containing 10% fetal bovine serum (Thermo Fisher Scientific) and 1% penicillin/streptomycin (Corning Inc.). Cells were maintained at 37 °C in a humidified atmosphere with 5% CO_2_/95% air. Recombinant mouse GREM2 protein was obtained from R&D Systems (USA). Recombinant mouse IL-6 protein was purchased from Sino Biological Inc. (China). Rabbit polyclonal GREM2 antibody was purchased from Abcam (UK). Anti-PPARγ, anti-C/EBPα, anti-FABP4, anti-vimentin, anti-slug, anti-twist1, and anti-β-actin were obtained from Cell Signaling Technology (USA). Epigallocatechin gallate (EGCG), metformin, and PKH26/PKH67 fluorescent cell linker kits were purchased from Sigma-Aldrich (USA).

### Establishment of stable cell line

3T3-L1 cell lines stably expressing either mock or Grem2 were established using a lentiviral transduction system. Briefly, mouse Grem2 lentiviral vector (EX-Mm06243-Lv122) was obtained from GeneCopoeia (USA). The lentiviruses were packaged in 293T cells using Lenti-Pac™ HIV expression packaging kit (GeneCopoeia). After 72 h transfection, the viral supernatant was collected, filtered and used for the transduction of 3T3-L1 cells in the presence of 8 μg/ml polybrene (Merck Millipore, USA). Stable cell lines were selected by 1 μg/ml puromycin (InvivoGen, USA).

### Differentiation of 3T3-L1 pre-adipocytes

3T3-L1 cells were cultured to reach 100% confluence in DMEM containing 10% BCS and then cultured for an additional 48 h. To initiate differentiation, the growth medium was removed and DMEM differentiation medium containing 10% FBS, 0.5 mM methylisobutylxanthine (Sigma-Aldrich, USA), 1 μM dexamethasone (Sigma-Aldrich), and 10 μg/ml insulin (Sigma-Aldrich) was added. After 48 h, the differentiation medium was replaced with DMEM adipocyte maintenance medium containing 10 μg/ml insulin, and the medium was changed every 48–72 h. When adipogenesis was observed in 3T3-L1 cells, the medium was replaced with fresh DMEM medium containing 10% FBS once every 2–3 days.

### Oil red O staining

3T3-L1 cells were differentiated and cultured for 9 days, and fat droplets were stain with Oil red O solution (Sigma-Aldrich). The dye was decolorized with 100% isopropyl alcohol and quantified at 500 nm absorbance.

### Western blot analysis

Standard sodium dodecyl sulfate–polyacrylamide gel electrophoresis (SDS-PAGE) and western blotting were used to analyze the expression of various proteins. Cells were lysed in the lysis buffer (Cell Signaling Technology) containing protease inhibitors and phosphatase inhibitors (Roche, Basel, Switzerland). The quantitative protein concentration was determined by BCA Protein Assay Kit (Thermo Fisher Scientific) and equal amounts of protein were loaded on 8–12% SDS-PAGE. Proteins were transferred to polyvinylidene difluoride membrane (Merck Millipore) and subjected to immunoblotting using various antibodies overnight at 4 °C, followed by further incubation with the secondary antibody (AbFrontier, Republic of Korea) at room temperature for 1 h. Visualization of protein bands was detected with Westsave Gold detection reagents (AbFrontier).

### 3D spheroid formation

3T3-L1 and MTV/TM-011 cells were labeled with green or red fluorescent cell linker (Sigma-Aldrich), respectively. The cells were mixed with each other in a 1:1 ratio [34] and seeded in 96-well low-adhesion plates (Cornning). To check the cell proliferation of the spheroids, the cell viability was analyzed using CellTiter-Glo® 3D cell viability assay (Promega, USA). For 3D spheroid invasion assays, 3T3-L1 and MTV/TM-011 cells were mixed with extracellular matrix and seeded in the 3D culture qualified 96 well spheroid formation plate (CULTREX). After 3-days incubation, the invasion matrix was added. The plate was incubated for additional 5–9 days and the invasion of spheroids was observed under the microscope. The obtained images were analyzed using software ImageJ described by the manufacturer.

### Reverse transcription-quantitative polymerase chain reaction (RT-qPCR)

Total RNA was isolated from cells using TRIzol® (Thermo Fisher Scientific). Reverse transcription of total RNA was performed using M-MLV reverse transcriptase (Enzynomics, Republic of Korea) according to the manufacturer’s protocol. Quantitative PCR (qPCR) was performed using TOP real™ qPCR 2X Pre-MIX (Enzynomics) and StepOnePlus Real-Time PCR (Thermo Fisher Scientific). Actb was used as internal references. Primer sequences are listed in the Additional file 1: Table S1.

### IL-6 enzyme-linked immunosorbent assay (ELISA) assay

After differentiation of 3T3-L1-mock or 3T3-L1-Grem2 cells for 9 days, conditioned medium was obtained from each cell line. Each conditioned medium was placed on an IL-6 ELISA microplate (Thermo Fisher Scientific) and incubated for 2 h. All procedures were performed according to the manufacturer's instructions.

### In vivo mouse model

Female BALB/c mice, 5 weeks of age (weight 18–20 ± 1–2 g) were purchased from Orient Bio Inc. (Republic of Korea). Mice were controlled in specific pathogen free conditions: 20–24 °C, 12/12 h of dark/light cycle, 60 ± 5% humidity, and plastic cage (4 mice/cage). For the orthotopic breast cancer mouse model, differentiated 3T3-L1-mock or 3T3-L1-Grem2 cells were mixed with MTV/TM-011 cells and inoculated into the fourth mammary fat pad of anesthetized mice by isoflurane inhalation. Both the volume of the primary tumors and the body weight of mice were measured twice a week. At the end of the experiment, mice were euthanized by CO inhalation and each tumor was removed.

### Immunofluorescence staining

Before staining of fixed paraffin-embedded tissues, we followed the standard protocol including the steps of deparaffinization, antigen retrieval, and permeabilization. For immunofluorescence, detection of primary antibodies was done using fluorescent conjugates of Alexa Fluor® 488 antibody (Thermo Fisher Scientific) along with ProLong® Gold Antifade Reagent with DAPI (Thermo Fisher Scientific).

### Statistical analysis

Data were expressed as the mean ± SD of results obtained from at least three independent experiments. Significant differences were determined by a Student’s *t*-test or one/two-way ANOVA. A *p* value of less than 0.05 was considered to be statistically significant.

## Results

### GREM2 inhibits adipogenesis in pre-adipocytes

**Fig. 1:**
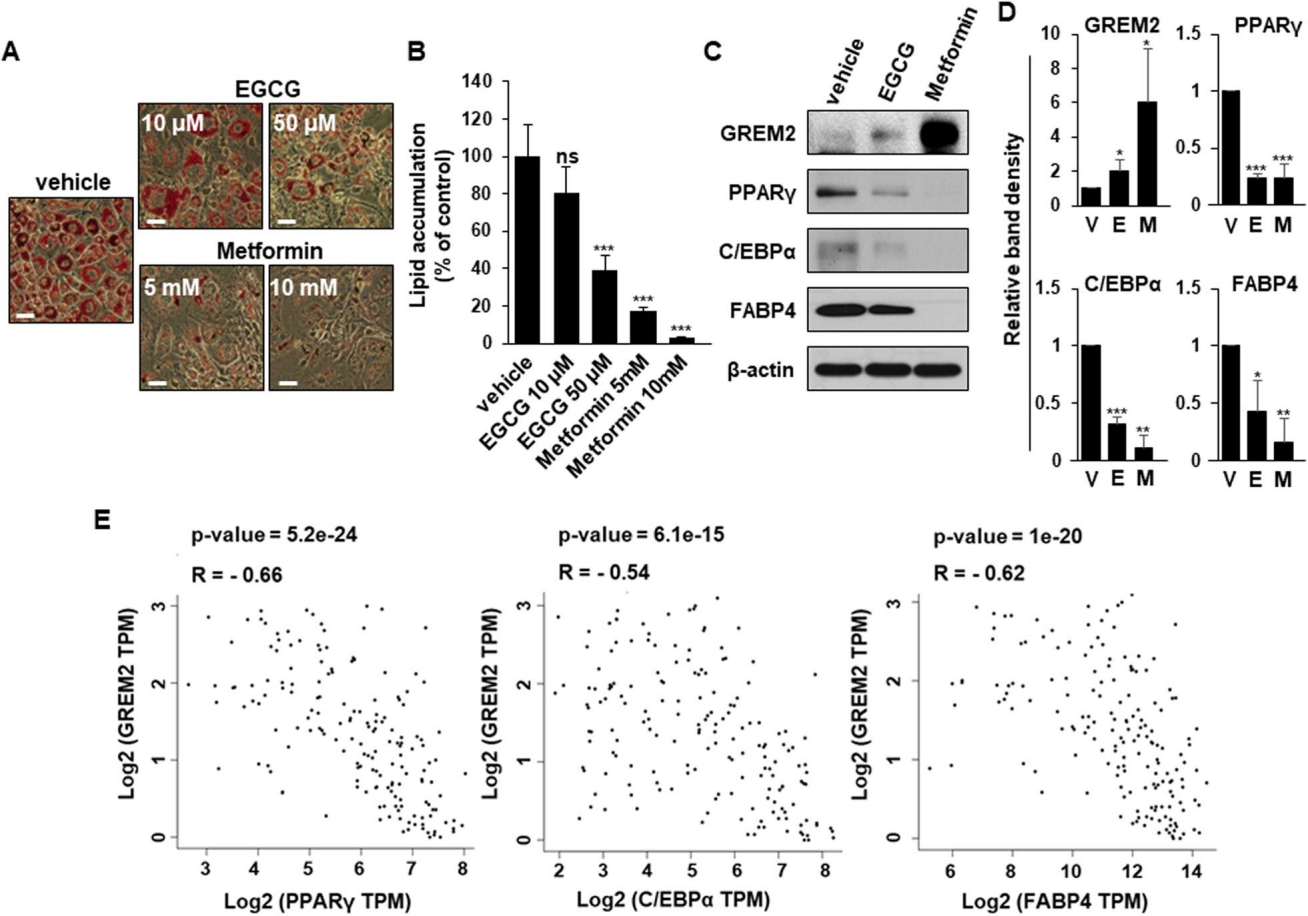
GREM2 expression is increased in adipocytes in which adipogenesis is inhibited by adipogenic inhibitors. **A**–**C** 3T3-L1 cells were cultured to confluence and differentiated using the standard induction cocktail with EGCG (10 or 50 μM) or metformin (5 or 10 mM) for 9 days. The effect of EGCG or metformin on lipid accumulation was measured by Oil red O staining (**A**). The bar graph represents relative lipid accumulation. Two-sided *t*-test. ***, *p* < 0.001, ns, not significant (**B**). On the 9th day of differentiation, proteins were isolated from adipocytes and the lysates were immunoblotted with the indicated antibodies (**C**). **D** The bar graphs represent relative band intensity. V: vehicle, E: EGCG, M: metformin. Two-sided *t*-test. *, *p* < 0.05; **, *p* < 0.01; ***, *p* < 0.001. **E** The correlation between GREM2 and adipogenic regulators in GTEx human breast tissues was investigated by using the web GEPIA. Correlation analysis was conducted using Spearman rank test

It has been reported that GREM2 can inhibit adipogenesis in pre-adipocytes [12]. To confirm the function of GREM2 as an adipogenic inhibitor, GREM2 expression levels in differentiated adipocytes with or without treatment with well-known adipogenic inhibitors such as epigallocatechin-gallate (EGCG) and metformin were compared. 3T3-L1 pre-adipocytes were cultured to confluence and differentiated using the standard induction cocktail with EGCG or metformin for 9 days. EGCG and metformin reduced the adipogenesis of 3T3-L1 cells, which was confirmed by Oil red O staining (Fig. 1a, b). Interestingly, expression levels of adipocyte differentiation regulators such as PPARγ, C/EBPα, and FABP4 were decreased, whereas the expression of GREM2 was increased in adipocytes treated with high concentrations of EGCG and metformin (Fig. 1c, d). Conflicting expression of these adipogenic regulators and GREM2 was more pronounced in metformin-treated cells, which showed significantly inhibited adipogenesis, than in EGCG-treated cells. To investigate correlations between GREM2 and adipogenic regulators such as PPARγ, C/EBPα, and FABP4, GTEx datasets from human breast tissues were obtained using the web server GEPIA [35]. As shown in Fig. 1e, correlation analysis revealed negative correlations of GREM2 with PPARγ (Spearman r = − 0.66, *p* = 5.2 × 10^−24^), C/EBPα (Spearman r = − 0.54, *p* = 6.1 × 10^−15^), and FABP4 (Spearman r = − 0.62, *p* = 1 × 10^−20^).

**Fig. 2:**
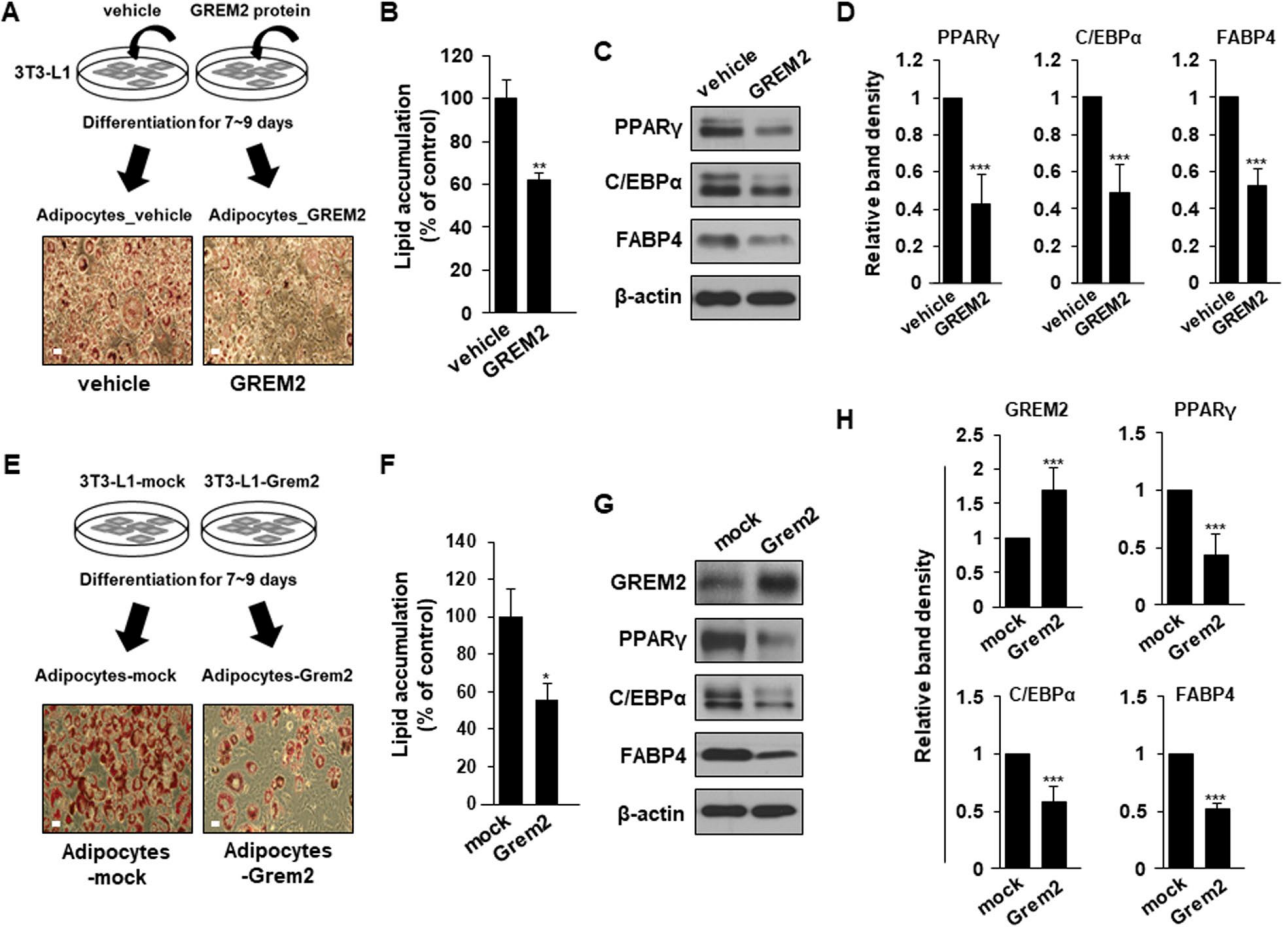
GREM2 inhibits adipogenesis in 3T3-L1 pre-adipocytes. **A** and **B** 3T3-L1 cells were cultured to confluence and differentiated using the standard induction cocktail with vehicle or GREM2 recombinant protein (50 ng/ml) for 9 days. The effect of vehicle or GREM2 protein on lipid accumulation was measured by Oil red O staining (**A**) and the bar graph represents relative lipid accumulation (**B**). **E** and **F** 3T3-L1-mock or 3T3-L1-Grem2 pre-adipocytes were cultured to confluence and differentiated using the standard induction cocktail for 9 days. The effect of mock or *Grem2* overexpression in pre-adipocytes on lipid accumulation was measured by Oil red O staining (**E**) and the bar graph represents relative lipid accumulation (**F**). **C** and **G** On the 9th day of differentiation, proteins were isolated from adipocytes and the lysates were immunoblotted with the indicated antibodies. **D** and **H** The bar graphs represent relative band intensity. Two-sided *t*-test. ***, *p* < 0.001

To determine whether GREM2 could directly inhibit 3T3-L1 adipogenesis, mouse recombinant GREM2 protein was used to treat 3T3-L1 cells for 9 days to induce differentiation. Cell morphology and Oil red O staining results indicated that cells differentiated by treatment with GREM2 protein accumulated significantly less lipids than vehicle-treated cells (Fig. 2a, b). In addition, expression levels of adipogenic regulators such as PPARγ, C/EBPα, and FABP4 were reduced in differentiated 3T3-L1 cells treated with GREM2 protein (Fig. 2c, d). Next, 3T3-L1 cell lines stably overexpressing mock or *Grem2* were established using a lentiviral transduction system. 3T3-L1-mock or 3T3-L1-Grem2 cells were cultured to confluence and differentiated into adipocytes (adipocytes-mock and adipocytes-Grem2) using the standard induction cocktail for 9 days. As shown in Fig. 2e and f, lipid accumulation was significantly inhibited in adipocytes-Grem2 cells compared to that in control cells. Expression levels of adipogenic regulators were also reduced in adipocytes-Grem2 cells (Fig. 2g, h). Together, these results indicate that GREM2 is an important regulator of adipogenesis by suppressing it.

### *Grem2*-overexpressing adipocytes inhibit proliferation and invasion of breast cancer cells

**Fig. 3:**
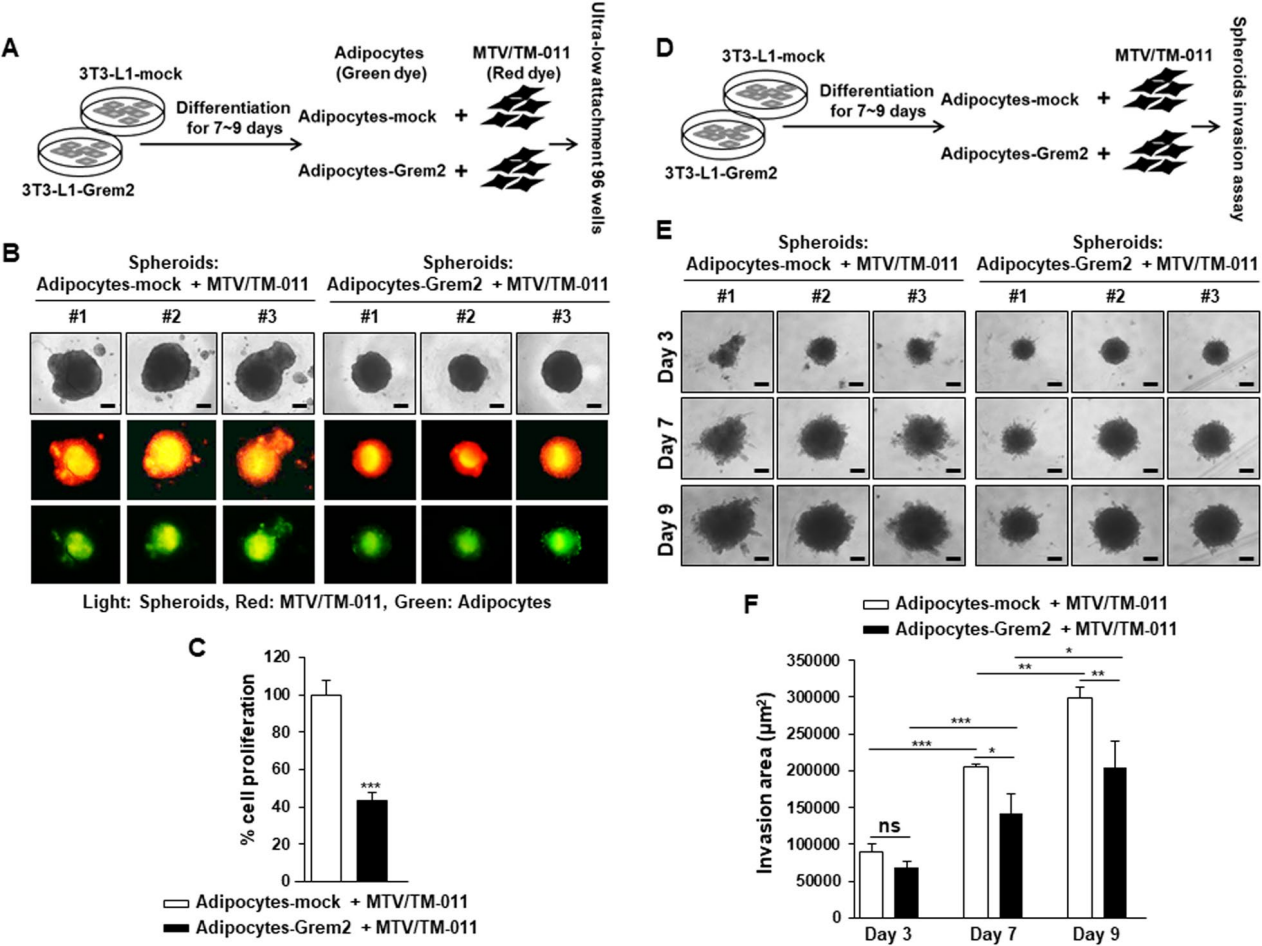
Adipocytes overexpressing *Grem2* inhibit the proliferation and invasion of breast cancer cells. **A**–**C** After differentiation of 3T3-L1-mock or 3T3-L1-Grem2 pre-adipocytes for 9 days, each cell line was mixed with MTV/TM-011 cells and seeded into ultra-low attachment 96 wells. Before mixing the cells, adipocytes were fluorescently stained green and breast cancer cells red (**A**). The growth of spheroids by 3D co-culture was observed under a fluorescence microscope (**B**) and their proliferation level was measured using the CellTiter-Glo® 3D Cell Viability Assay (**C**). Scale bar = 100 µm. Two-sided *t*-test. ***, *p* < 0.001. **D**–**F** After differentiation of 3T3-L1-mock or 3T3-L1-Grem2 pre-adipocytes for 9 days, each cell line was mixed with MTV/TM-011 cells and performed 3D invasion assay (**D**, **E**). The invasion area was measured using the Image J (**F**). Scale bar = 100 µm. Two-way ANOVA. *, *p* < 0.05; **, *p* < 0.01; ***, *p* < 0.001; ns, not significant

Adipogenesis is associated with obesity, which in turn may increase the risk and aggressiveness of breast cancer [36]. We used a 3D co-culture system of adipocytes and breast cancer cells to investigate the effect of GREM2-suppressed adipogenesis on breast cancer cells. 3T3-L1-mock and 3T3-L1-Grem2 cells were differentiated into adipocytes (adipocytes-mock and adipocytes-Grem2) for 9 days and then mixed with breast cancer MTV/TM-011 cells at the same ratio to form 3D spheroids in ultra-low attachment plates (Fig. 3a). Cell membranes of adipocytes and breast cancer cells were stained with fluorescent dyes of different colors (red: breast cancer cells, green: adipocytes) to confirm that these cells were well mixed within the spheroid. As shown in Fig. 3b, sizes of spheroids formed by co-culture of MTV/TM-011 cells and adipocytes-Grem2 were smaller than those of spheroids formed by co-culture of MTV/TM-011 cells and adipocytes-mock. CellTiter-Glo® 3D Cell Viability Assay results also showed that the proliferation of spheroids formed by co-culture of MTV/TM-011 cells and adipocytes-Grem2 was significantly reduced compared to that of the control group (Fig. 3c).

Next, we performed a co-spheroid invasion assay to determine the effect of *Grem2-*overexpressing adipocytes on invasion ability of breast cancer cells (Fig. 3d). After culturing spheroids with invasion matrix, the invasion area of each spheroid was measured on the 3rd, 7th, and 9th days. As a result, it was confirmed that the invasion area of spheroids formed with MTV/TM-011 cells and adipocytes-Grem2 was significantly suppressed compared to that of spheroids formed with MTV/TM-011 cells and adipocytes-mock (Fig. 3e, f). These results suggest that *Grem2*-overexpressing adipocytes can inhibit the proliferation and invasiveness of breast cancer cells.

### Expression of adipokines is lower in *Grem2*-overexpressing adipocytes than in control cells

**Fig. 4:**
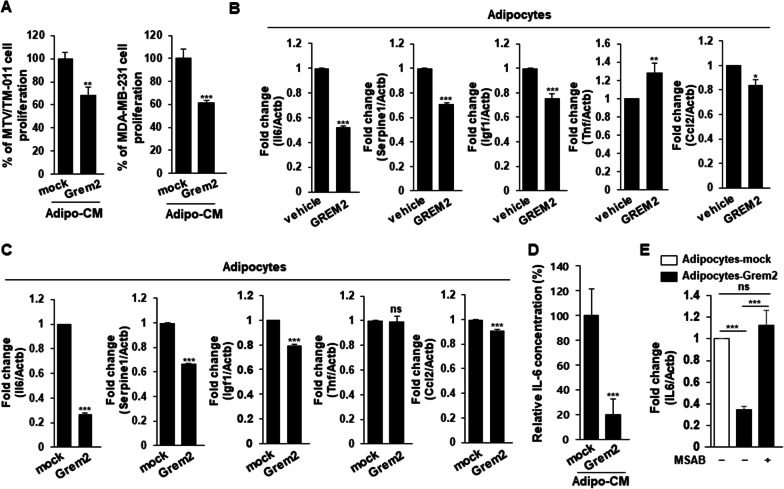
IL-6 expression is reduced in adipocytes overexpressing *Grem2*. **A** 3T3-L1-mock or 3T3-L1-Grem2 cells were differentiated into adipocytes, and each CM was obtained from day 7 to day 9 when adipogenesis was observed. MTV/TM-011 or MDA-MB-231 cells were seeded in 96 well plates and incubated with each CM for 72 h, followed by MTS assay. **B** 3T3-L1 cells were differentiated using the standard induction cocktail with vehicle or GREM2 recombinant protein (50 ng/ml) for 9 days. RNA was isolated from each cell line and the expression of the indicated genes was analyzed by qPCR. **C** 3T3-L1-mock or 3T3-L1-Grem2 cells were differentiated using the standard induction cocktail for 9 days. RNA was isolated from each cell line and the expression of the indicated genes was analyzed by qPCR. **D** 3T3-L1-mock or 3T3-L1-Grem2 cells were differentiated into adipocytes, and IL-6 content was examined in each adipocyte CM obtained on the 9^th^ day of differentiation. Two-sided *t*-test. *, *p* < 0.05; **, *p* < 0.01; ***, *p* < 0.001; ns, not significant. **E** Adipocytes-mock or adipocytes-Grem2 cells were treated with or without MSAB (10 μM) for 24 h. RNA was isolated from each cell line and the expression of the indicated genes was analyzed by qPCR. Two-way ANOVA. ***, *p* < 0.001; ns, not significant

Various adipokines secreted from adipocytes are known to promote cancers including breast cancer [37–39]. We hypothesized that adipocytes overexpressing *Grem2* could inhibit breast cancer cell proliferation and invasion by suppressing certain adipokines. First, breast cancer cells were directly treated with conditioned medium (CM) obtained from differentiated pre-adipocytes. Its effect on proliferation of breast cancer cells was investigated. As a result, it was confirmed that the proliferation of breast cancer cells (MTV/TM-011 or MDA-MB-231) treated with the CM obtained from adipocytes-Grem2 was significantly reduced compared to that of cells treated with CM obtained from adipocytes-mock (Fig. 4a). Next, we investigated mRNA levels of several adipokines using qRT-PCR to determine which adipokines were suppressed in *Grem2*-overexpressing adipocytes. After differentiation of 3T3-L1 cells by treatment with vehicle or mouse recombinant GREM2 protein, RNA was isolated from each adipocyte and mRNA expression levels of *Il6*, *Serpine1*, *Igf1*, *Tnf*, and *Ccl2* were determined. As shown in Fig. 4b, the expression levels of all adipokines except *Tnf* were significantly decreased in adipocytes differentiated by GREM2 protein treatment. Consistent with these results, the expression of several adipokines, including *Il6,* in adipocytes differentiated from 3T3-L1-Grem2 cells was significantly decreased compared to that in the control group (Fig. 4c). In addition, IL-6 ELISA analysis revealed that the concentration of IL-6 present in the culture medium of *Grem2*-overexpressing adipocytes was significantly reduced compared to that in the control group (Fig. 4d). Based on previous studies showing that overexpression of *Grem2* in adipocytes increases Wnt/β-catenin signaling, we further tested whether GREM2 suppresses IL-6 expression by increasing Wnt/β-catenin activity in adipocytes. As shown in the Fig. 4e, IL-6 expression was decreased in adipocytes-Grem2 compared to adipocytes-mock and again significantly increased when co-treated with MSAB, a Wnt/β-catenin inhibitor.

Various studies have reported that cytokines, including IL-6, are expressed in cancer cells themselves [40–44]. Recently, it was confirmed that breast cancer cells are secretory cells capable of producing various cytokines using the 41 cytokine MILLIPLEX assay [45]. Therefore, we checked whether *Grem2* overexpression in adipocytes also affected the reduction of IL-6 expression in breast cancer cells. After culturing adipocytes-mock or adipocytes-Grem2 on the transwells and MTV/TM-011 breast cancer cells on the bottom for 48 h, changes in IL-6 mRNA expression in breast cancer cells were examined. Interestingly, the expression of IL-6 in breast cancer cells was significantly reduced by co-culture of adipocytes-Grem2 (Additional file 2: Fig. S1). Taken together, these results suggest that *Grem2*-overexpressing adipocytes have the potential to suppress the expression of cytokines, including IL-6, in adipocytes or breast cancer cells, which may contribute to the inhibition of breast cancer progression.

### IL-6 affects the restoration of the proliferative and invasion abilities of breast cancer cells reduced by *Grem2*-overexpressing adipocytes

**Fig. 5:**
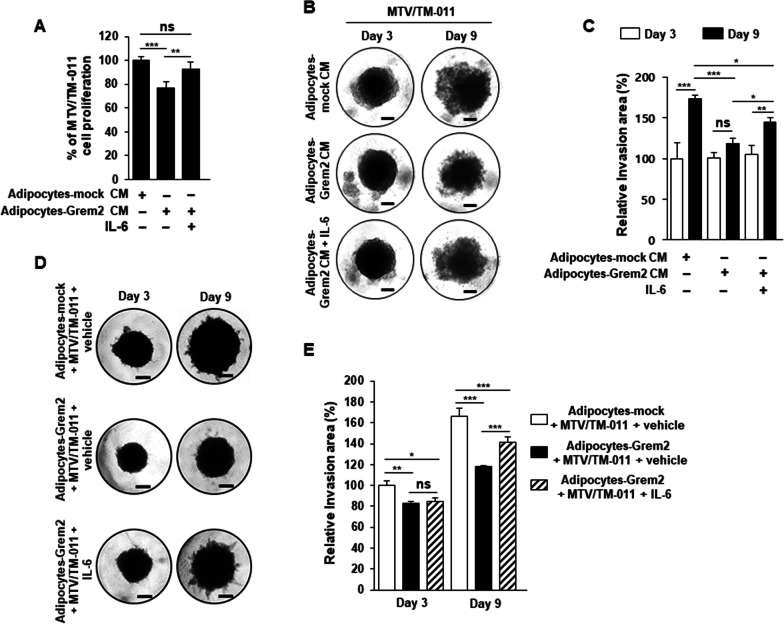

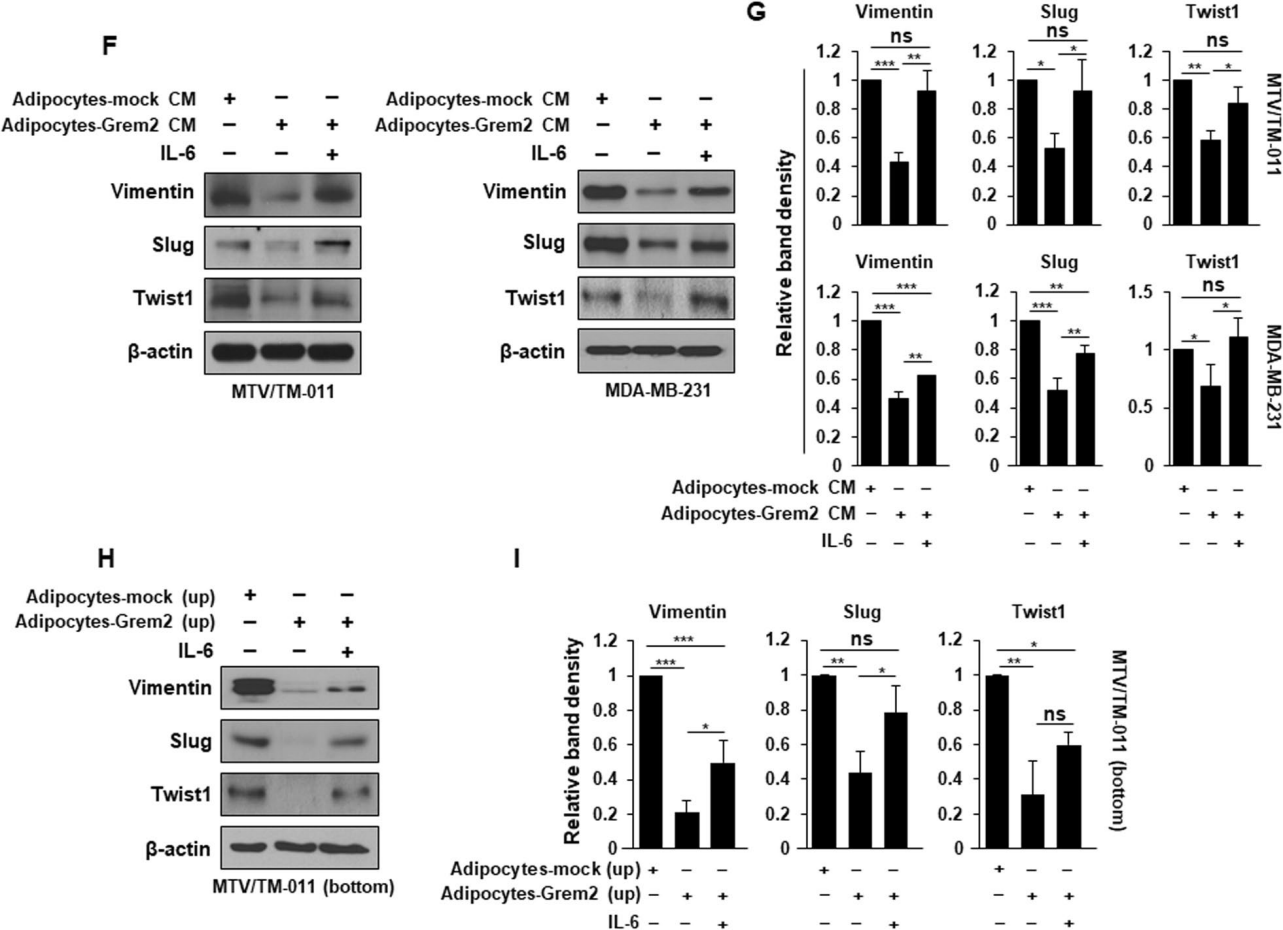
IL-6 restores the ability of breast cancer cells to proliferate and invade, which was inhibited by adipocytes overexpressing *Grem2*. **A** MTV/TM-011 cells were seeded in a 96-well plate and treated with each adipocyte CM or IL-6 recombinant protein (25 ng/ml) for 72 h, followed by MTS assay. **B** MTV/TM-011 cells were treated with each adipocyte CM or IL-6 recombinant protein (25 ng/ml), and then 3D invasion analysis was performed. Scale bar = 100 µm. **D** After differentiation of 3T3-L1-mock or 3T3-L1-Grem2 into adipocytes for 9 days, 3D invasion assay was performed by mixing adipocytes-mock or adipocytes-Grem2 with MTV/TM-011 cells. Scale bar = 100 µm. **C** and **E** The relative invasion area was measured using the Image J. **F** MTV/TM-011 or MDA-MB-231 cells were treated with each adipocyte CM or IL-6 recombinant protein (25 ng/ml) for 72 h. The lysates were immunoblotted with the indicated antibodies. **H** Each adipocyte and MTV/TM-011 cells were co-cultured for 72 h in a transwell system (upper chamber: adipocytes, bottom: MTV/TM-011) with or without IL-6 recombinant protein (25 ng/ml). The lysates of MTV/TM-011 cells were immunoblotted with the indicated antibodies. **G** and **I** The bar graphs represent relative band intensity. Two-way ANOVA. *, *p* < 0.05; **, *p* < 0.01; ***, *p* < 0.001; ns, not significant

Next, whether reduced expression and secretion of IL-6 in *Grem2*-overexpressing adipocytes had a direct effect on the proliferation of breast cancer cells was determined. MTV/TM-011 breast cancer cells were treated with CM from mock- or *Grem2-*overexpressing adipocytes. CM obtained from *Grem2*-overexpressing adipocytes decreased the proliferation of breast cancer cells compared to the control. Such decrease was significantly restored by IL-6 recombinant protein treatment (Fig. 5a). To examine effects of *Grem2*-overexpressing adipocytes and IL-6 protein on invasive capacity of breast cancer cells, we performed a 3D invasion assay. As shown in Fig. 5b and c, the invasive capacity of MTV/TM-011 cells was decreased by CM obtained from *Grem2*-overexpressing adipocytes compared to the control. Such decrease was again significantly rescued by IL-6 treatment. These results were consistent with results of the invasion assay by 3D co-culture of breast cancer cells and adipocytes overexpressing *Grem2*. Co-spheroids of MTV/TM-011 cells and *Grem2*-overexpressing adipocytes showed decreased invasive ability compared to the control. Such decrease was significantly restored by further treatment with IL-6 protein (Fig. 5d, e). The invasion ability of breast cancer cells, which was reduced by adipocyte-Grem2, was greatly restored by IL-6 treatment alone, but additional administration of Serpine1, another cytokine reduced by adipocyte-Grem2, had a greater effect on recovering cancer cell invasion (Additional file 3: Fig. S2).

To further confirm the effects of *Grem2*-overexpressing adipocytes and IL-6 on the invasion ability of breast cancer cells, expression levels of key proteins related to epithelial-mesenchymal transition (EMT) and cancer cell invasion were examined. Two breast cancer cell lines MTV/TM-011 and MDA-MB-231 were treated with CM obtained from adipocytes-mock or adipocytes-Grem2. As a result, expressions levels of vimentin, slug, and twist1 were all decreased in breast cancer cells by the CM obtained from adipocytes-Grem2. However, expression levels of these proteins were increased again by combined treatment with adipocytes-Grem2 CM and IL-6 (Fig. 5f, g). Next, adipocytes and breast cancer cells were indirectly co-cultured through a transwell system. As a result of co-culture of MTV/TM-011 cells and adipocytes-Grem2, protein expression levels of vimentin, slug, and twist1 in MTV/TM-011 cells were significantly decreased compared to those of co-culture with adipocytes-mock. Incubation with IL-6 and adipocytes-Grem2 also increased expression levels of these proteins in MTV/TM-011 cells (Fig. 5h, i). These results suggest that adipokines, especially IL-6, are reduced in adipocytes overexpressing *Grem2*, which has the effect of suppressing the proliferation and invasion of breast cancer cells.

### Proliferative and lung metastatic abilities of breast cancer cells in vivo are decreased by *Grem2*-overexpressing adipocytes but increased by IL-6 treatment

**Fig. 6:**
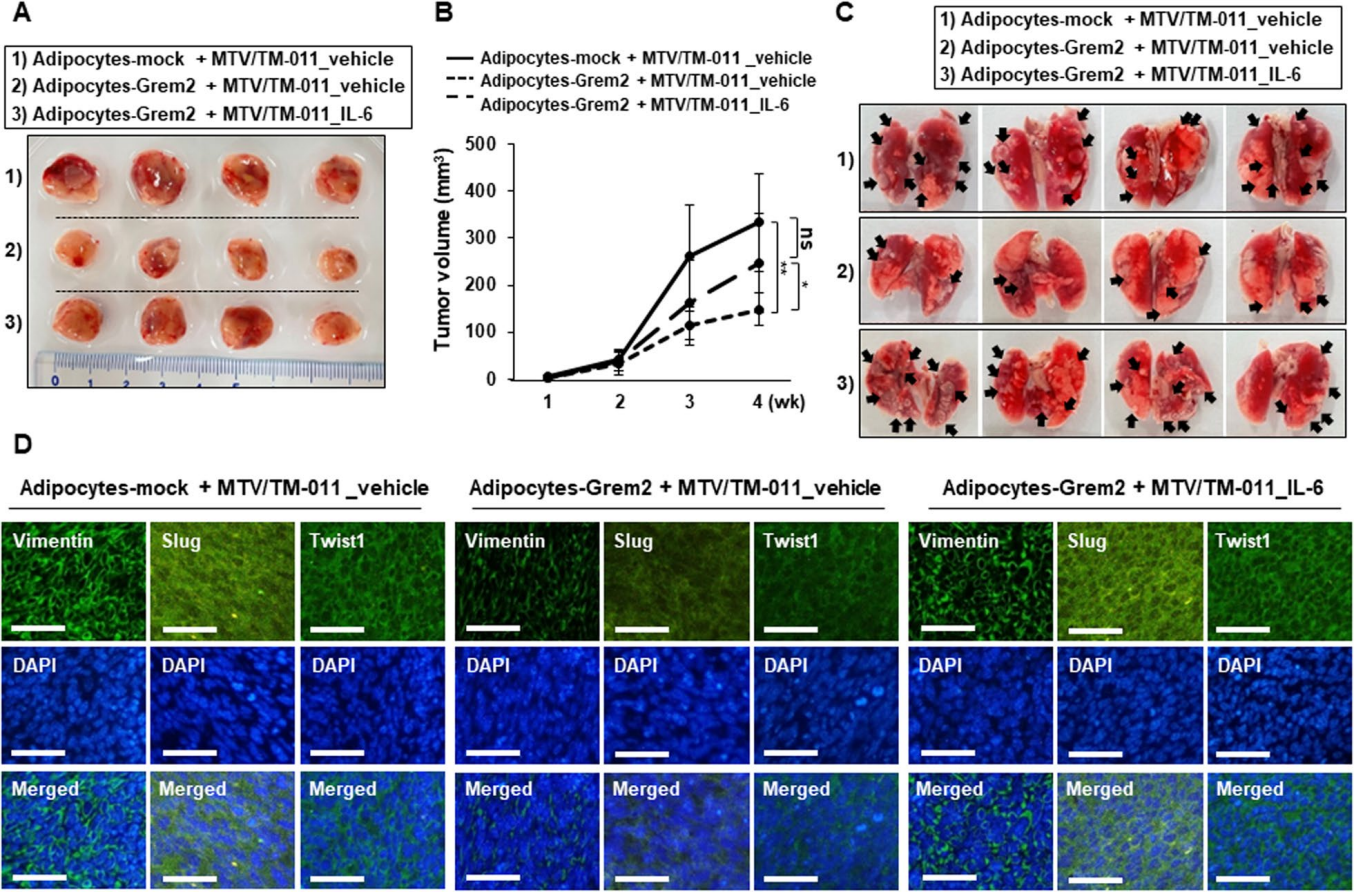
IL-6 restores breast cancer proliferation and lung metastasis inhibited by adipocytes overexpressing *Grem2*. **A**–**C** After differentiation of 3T3-L1-mock or 3T3-L1-Grem2 into adipocytes for 9 days, adipocytes-mock or adipocytes-Grem2 were mixed with MTV/TM-011 cells and injected into mammary fat pads of mice. Representative images of primary tumors (**A**), tumor volume (**B**), and lung metastatic foci (**C**). Black arrowheads indicate prominent lung metastatic foci. **D** Representative immunofluorescence images of vimentin, slug, and twist1 in tumors. Scale bar = 100 μm. Two-way ANOVA. *, *p* < 0.05; **, *p* < 0.01; ns, not significant

To investigate the role of GREM2 in breast cancer proliferation and metastasis, 3T3-L1-mock or 3T3-L1-Grem2 cells were differentiated into adipocytes, mixed with MTV/TM-011 cells, and orthotopically implanted into mammary fat pads of mice. Mice injected with MTV/TM-011 cells and adipocytes-Grem2 developed smaller primary tumors than mice injected with MTV/TM-011 cells and adipocytes-mock, resulting in a dramatic reduction in tumor volume [mean ± SD (mm^3^): 333.07 ± 103.87 (MTV/TM-011 + adipocytes-mock_vehicle) vs. 148.78 ± 34.16 (MTV/TM-011 + adipocytes-Grem2_vehicle), six mice/each group]. Interestingly, intraperitoneal administration of recombinant IL-6 protein in mice injected with MTV/TM-011 cells and adipocytes-Grem2 accelerated primary tumor growth compared to vehicle-treated group [mean ± SD (mm^3^): 148.78 ± 34.16 (MTV/TM-011 + adipocytes-Grem2_vehicle) vs. 249.55 ± 97.8 (MTV/TM-011 + adipocytes-Grem2_IL-6], six mice in MTV/TM-011 + adipocytes-Grem2_vehicle; five mice in MTV/TM-011 + adipocytes-Grem2_IL-6) (Fig. 6a, b). Consistent with the growth outcome of the primary tumor, mice injected with MTV/TM-011 cells and adipocytes-Grem2 had less lung metastases than mice injected with MTV/TM-011 cells and adipocytes-mock. Additionally, intraperitoneal administration of recombinant IL-6 protein in mice injected with MTV/TM-011 cells and adipocytes-Grem2 increased the number of metastatic lung lesions compared to the vehicle-treated group (Fig. 6c and Additional file 4: Fig. S3). To further confirm effects of *Grem2*-overexpressing adipocytes and IL-6 on breast cancer progression in vivo, levels of vimentin, slug, and twist1 in primary tumors from each group were analyzed by immunofluorescence staining. As shown in Fig. 6d and Additional file 5: Fig. S4, expression levels of these proteins were significantly decreased in mice injected with MTV/TM-011 cells and adipocytes-Grem2 compared to those in mice injected with MTV/TM-011 cells and adipocytes-mock. In addition, when IL-6 was administered to mice injected with MTV/TM-011 cells and adipocytes-Grem2, expression levels of these proteins were increased again. Taken together, these results indicate that overexpressing *Grem2* in adipocytes can reduce breast cancer cell proliferation and lung metastasis in vivo and IL-6 can reverse these effects. These results suggest that decreased IL-6 secretion due to overexpression of *Grem2* in adipocytes contributes to suppressing breast cancer progression.

## Discussion

Since adipogenesis causes various diseases such as obesity and cancer, various compounds capable of suppressing it have been reported. In particular, EGCG, a green tea extract, and metformin, a hypoglycaemic drug, can inhibit adipogenesis and lipid accumulation of pre-adipocytes [46–49]. In this study, it was confirmed that when pre-adipocytes were treated with metformin, adipocyte differentiation was inhibited, while GREM2 expression was significantly increased. Together with these results, the inhibition of adipogenesis by GREM2 itself clearly showed that GREM2 could act as an adipogenic inhibitor. The present study demonstrates that GREM2 can inhibit adipogenesis and that adipocytes overexpressing *Grem2* play an important role in inhibiting breast cancer cell growth, migration, and metastasis.

The tumor microenvironment composed of various cell types and extracellular matrix proteins provides favorable conditions for the growth and survival of cancer cells. Therefore, it is important to consider the tumor microenvironment in order to effectively inhibit tumor proliferation and metastasis [50, 51]. Adipose tissue is a representative stromal tissue of the breast. Cancer-related adipocytes have been reported to play a role in promoting breast cancer in the breast cancer microenvironment. Our results suggest that inhibition of adipogenesis in adipocytes might affect the breast cancer microenvironment and inhibit the progression of breast cancer. In this study, the expression of several adipokines was significantly suppressed in adipocytes overexpressing *GREM2* compared to those in control cells. In particular, inhibition of the adipokine IL-6 by GREM2 had an effect in suppressing proliferation, migration, and metastasis of breast cancer cells in the breast cancer microenvironment. In addition to adipocytes, fibroblasts are also one of the major stromal cells constituting the breast [52–54]. Although the effect of GREM2 on cancer-associated fibroblasts in the breast cancer microenvironment is still unknown, exosomal miR-423-5p secreted by cancer-associated fibroblasts in prostate cancer is known to be able to promote chemotherapy resistance by inhibiting GREM2 [55]. Thus, further studies are needed to examine the relationship between GREM2 and cancer-associated fibroblasts in breast cancer.

This study showed that the expression and production of several adipokines were suppressed in *Grem2*-overexpressing adipocytes. One of the transcription factors that regulates the expression of the representative adipokine IL-6 is NF-κB [56, 57]. Wnt/β-catenin is known to negatively regulate the NF-κB signaling pathway [58, 59]. In human chondrocytes, Wnt-3A stimulation can suppress mRNA expression of IL-6 induced by NF-κB/IL-1β [60]. Activation of β-catenin also reduced NF-κB activity and the expression of its target gene IL-6 [61]. It has been reported that β-catenin can inhibit IL-6 transcription and expression in human astrocytes [62, 63]. C/EBP family members are also transcription factors involved in the expression of several adipokines, including IL-6, and are known to be negatively regulated by Wnt/β-catenin. C/EBPα, C/EBPβ and C/EBPδ are all involved in adipogenesis, and the Wnt/β-catenin pathway eventually inhibits them to suppress adipogenesis [64]. In addition, Wnt signaling suppressed C/EBPα and PPARγ to stimulate osteoblastogenesis of mesenchymal precursors [65]. Interestingly, GREM2 is known to inhibit adipogenesis of pre-adipocytes and adipose-derived stromal/stem cells by activating Wnt/β-catenin signaling [12, 13]. We confirmed that reduced IL-6 expression in *Grem2*-overexpressing adipocytes was restored when treated with a Wnt/β-catenin inhibitor. This shows that the increase in Wnt/β-catenin signaling by GREM2 is involved in the decrease in IL-6 expression. Although a direct relationship between GREM2 and IL-6 has not been reported yet, our results suggest that overexpression of *GREM2* in adipocytes might inhibit IL-6 expression through activation of Wnt/β-catenin signaling. This phenomenon may be related to decreased activity of NF-κB or C/EBP transcription factors due to increased Wnt/β-catenin signaling.

As an adipogenic inhibitor, GREM2 could be developed as a target for obesity or a therapeutic means to inhibit breast cancer progression. This study suggests that reduced expression of several adipokines in adipocytes overexpressing *GREM2* may contribute to breast cancer suppression. Additionally, our study suggests that GREM2, a type of cytokine, can be secreted when overexpressed in adipocytes and may directly contribute to the suppression of breast cancer independently of adipokines. The mechanism by which GREM2 directly affects breast cancer cells requires further detailed studies. Recently, research on gene therapy for various diseases has been actively conducted. One of the most frequently used techniques is inserting a gene of interest or healthy gene into a vector such as a plasmid, nanostructure, or virus [66, 67]. TP53 is a representative tumor suppressor involved in cell cycle arrest, apoptosis, and DNA damage repair. Gene therapy using adenoviral p53 has shown anti-tumor effects in pre-clinical and clinical studies [68, 69]. In this context, further studies are needed to utilize GREM2 for gene therapy or to discover small molecules that can increase GREM2 expression in adipocytes to effectively treat breast cancer.

## Conclusion

In conclusion, we, for the first time, report that *GREM2* overexpression in adipocytes can inhibit breast cancer proliferation and metastasis. GREM2 inhibited adipogenesis and the production of the adipokines, including IL-6, in adipocytes, which likely makes an important contribution to the inhibition of breast cancer progression. In addition, it is possible that GREM2 itself, secreted from *GREM2*-overexpressing adipocytes, has an effect on breast cancer suppression independently of adipokines. GREM2 can be an effective therapeutic target for treating various adipocyte-associated carcinomas. Our findings suggest that overexpressing *GREM2* in adipocytes is a novel therapeutic approach to effectively inhibit breast cancer proliferation and metastasis.

### Supplementary Information

**Additional file 1: Table S1.** qRT-PCR primer sequences.

**Additional file 2: Fig. S1.** Effect of adipocytes-Grem2 on IL-6 expression in breast cancer cells. Each adipocyte and MTV/TM-011 cells were co-cultured for 48 h in a transwell system (upper chamber: adipocytes, bottom: MTV/TM-011). RNA was isolated from MTV/TM-011 cells and the expression of the indicated genes was analyzed by qPCR. Two-sided t-test. **, *p* < 0.01.

**Additional file 3: Fig. S2.** **A** MTV/TM-011 cells were treated with each adipocyte CM, IL-6 recombinant protein (25 ng/ml) and/or Serpine1 recombinant protein (25 ng/ml) and 3D invasion analysis was performed. Scale bar = 100 µm. **B** The relative invasion area was measured using the Image J. Two-way ANOVA. *, *p* < 0.05; ***, *p* < 0.001; *ns* not significant.

**Additional file 4: Fig. S3.** Number of metastatic lung tumor nodules. The average number of lung metastatic nodules in each group was confirmed through H&E-stained slides of four representative lungs from each group. Two-way ANOVA. *, *p* < 0.05; *ns* not significant.

**Additional file 5: Fig. S4.** Quantification of fluorescence images for Figure 6D. Based on the expression of the corresponding DAPI, the expression levels of vimentin, slug, or twist1 were determined and analyzed in three independent tissues. Two-way ANOVA. *, *p* < 0.05; **, *p < 0.01*; ***, *p* < 0.001; *ns* not significant.

## Data Availability

Available upon request to corresponding author (sappark@sch.ac.kr).
