# Supplementary material for: The inhibitory effect of Gremlin-2 on adipogenesis suppresses breast cancer cell growth and metastasis
Source: Breast Cancer Res. 2023 Oct 25;25:128. doi: 10.1186/s13058-023-01732-2 (PMC10599028; doi:10.1186/s13058-023-01732-2)
Supplement: Supplementary file 1 — Additional file 1: Table S1. qRT-PCR primer sequences. [file 13058_2023_1732_MOESM1_ESM.docx]

**Supplementary Table 1. qRT-PCR primer sequences**

| **Genes** | **Forward** | **Reverse** |
| --- | --- | --- |
| Actb | GGCTGTATTCCCCTCCATCG | CCAGTTGGTAACAATGCCATGT |
| Il6 | TAGTCCTTCCTACCCCAATTTCC | TTGGTCCTTAGCCACTCCTTC |
| Serpine1 | TTCAGCCCTTGCTTGCCTC | ACACTTTTACTCCGAAGTCGGT |
| Igf1 | CTGGACCAGAGACCCTTTGC | GGACGGGGACTTCTGAGTCTT |
| Tnf | GACGTGGAACTGGCAGAAGAG | TTGGTGGTTTGTGAGTGTGAG |
| Ccl2 | TTAAAAACCTGGATCGGAACCAA | GCATTAGCTTCAGATTTACGGGT |
