# Supplementary figures and images for: The inhibitory effect of Gremlin-2 on adipogenesis suppresses breast cancer cell growth and metastasis
Source: Breast Cancer Res. 2023 Oct 25;25:128. doi: 10.1186/s13058-023-01732-2 (PMC10599028; doi:10.1186/s13058-023-01732-2)

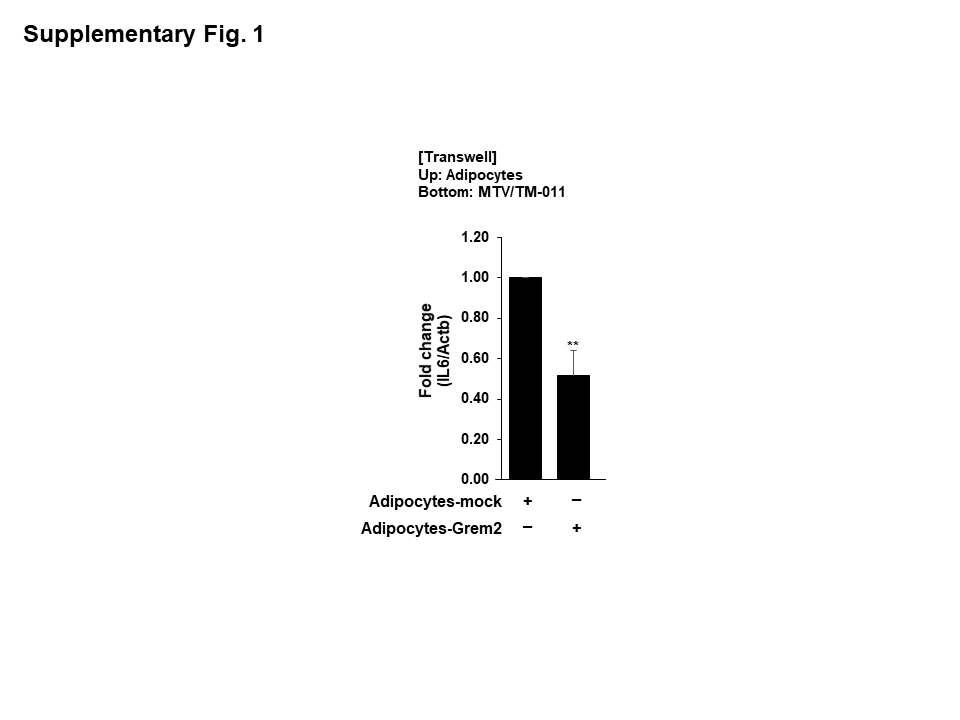

Supplement: Supplementary file 2 — Additional file 2: Fig. S1. Effect of adipocytes-Grem2 on IL-6 expression in breast cancer cells. Each adipocyte and MTV/TM-011 cells were co-cultured for 48 h in a transwell system (upper chamber: adipocytes, bottom: MTV/TM-011). RNA was isolated from MTV/TM-011 cells and the expression of the indicated genes was analyzed by qPCR. Two-sided t-test. **, p < 0.01. [file 13058_2023_1732_MOESM2_ESM.tif]

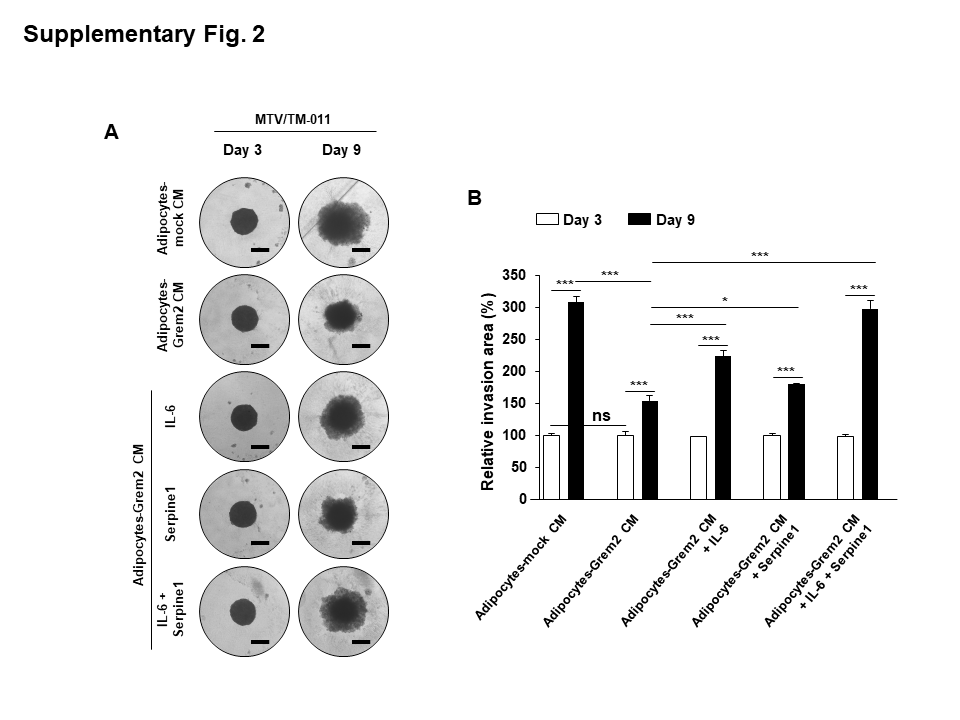

Supplement: Supplementary file 3 — Additional file 3: Fig. S2. A MTV/TM-011 cells were treated with each adipocyte CM, IL-6 recombinant protein (25 ng/ml) and/or Serpine1 recombinant protein (25 ng/ml) and 3D invasion analysis was performed. Scale bar = 100 µm. B The relative invasion area was measured using the Image J. Two-way ANOVA. *, p < 0.05; ***, p < 0.001; ns not significant. [file 13058_2023_1732_MOESM3_ESM.tif]

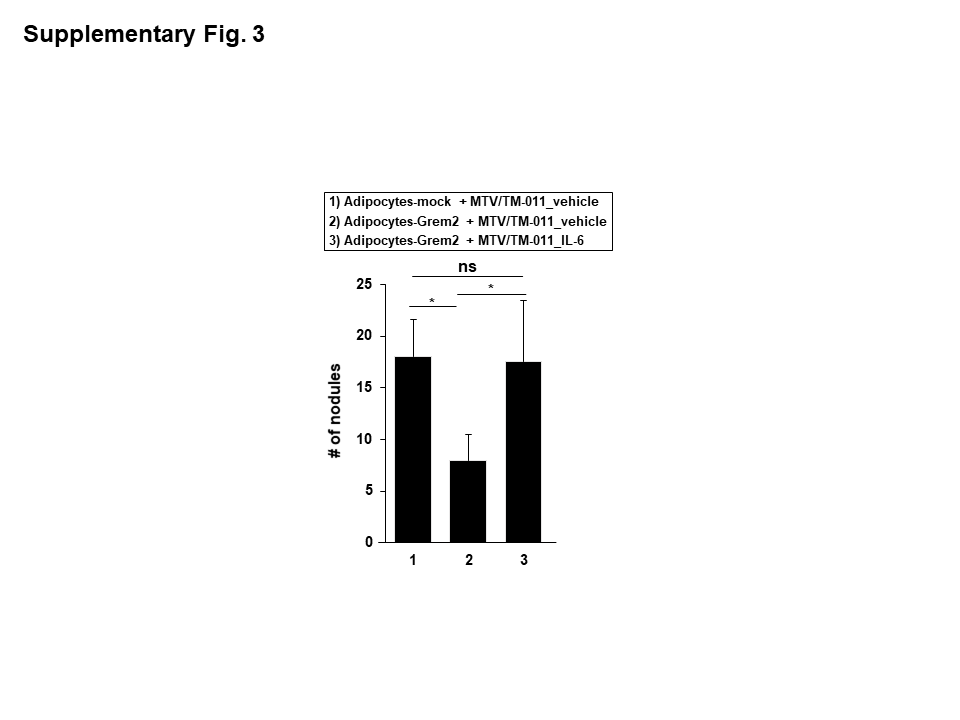

Supplement: Supplementary file 4 — Additional file 4: Fig. S3. Number of metastatic lung tumor nodules. The average number of lung metastatic nodules in each group was confirmed through H&E-stained slides of four representative lungs from each group. Two-way ANOVA. *, p < 0.05; ns not significant. [file 13058_2023_1732_MOESM4_ESM.tif]

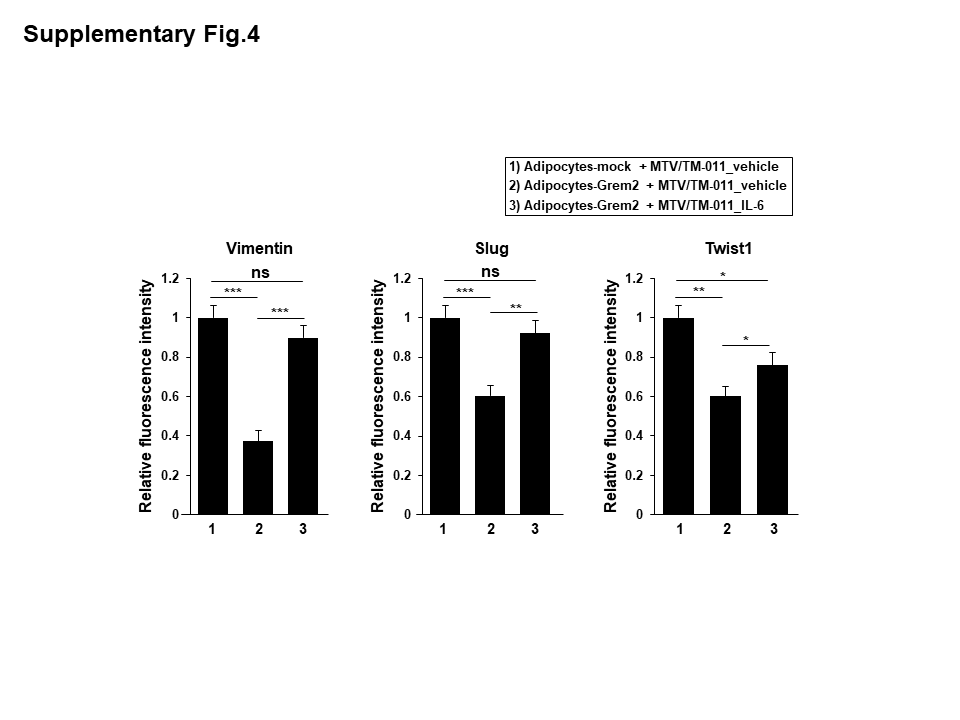

Supplement: Supplementary file 5 — Additional file 5: Fig. S4. Quantification of fluorescence images for Figure 6D. Based on the expression of the corresponding DAPI, the expression levels of vimentin, slug, or twist1 were determined and analyzed in three independent tissues. Two-way ANOVA. *, p < 0.05; **, p < 0.01; ***, p < 0.001; ns not significant. [file 13058_2023_1732_MOESM5_ESM.tif]
